# Rapid review and meta-analysis of the effectiveness of personal protective equipment for healthcare workers during the COVID-19 pandemic

**DOI:** 10.1016/j.puhip.2022.100280

**Published:** 2022-06-13

**Authors:** Daniela Schoberer, Selvedina Osmancevic, Lea Reiter, Nina Thonhofer, Manuela Hoedl

**Affiliations:** Institute of Nursing Science, Medical University of Graz, Universitaetsplatz4/3, 8010, Graz, Austria

**Keywords:** COVID-19, Personal protective equipment, Face mask, Health care worker

## Abstract

**Objectives:**

Healthcare workers (HCWs) worldwide have and are using personal protective equipment (PPE) as COVID-19 prevention measures, including gloves, gowns, goggles, masks and hand hygiene. Although several reviews have been published on the effectiveness of PPE, these often include studies on other inflectional diseases. This is problematic, because these diseases differ with regard to, e.g. the transmissibility and viral loads in the days after infection. Therefore, we assessed the effectiveness of PPE to protect HCWs from COVID-19 infections.

**Design:**

Rapid review of literature.

**Methods:**

We followed a practical guide to conduct the rapid review based on a protocol established by the Cochrane Rapid Reviews Methods Group. Meta-analyses have been conducted to synthesize the results. The confidence in the evidence was determined using the GRADE method.

**Results:**

We found 461 reviews and 208 primary studies, of which 16 systematic reviews included 11 observational studies of interest. Wearing PPE conferred significant protection against infection with COVID-19 as opposed to not wearing adequate PPE. Overall, the review results show that wearing face masks can significantly protect HCWs from infection. We found no effects for wearing gloves and gowns. Practicing thorough hand hygiene and having proper PPE, as compared to lacking proper PPE, showed a protective but not statistically significant effect. No studies reported the side effects of wearing PPE or acceptance rates.

**Conclusion:**

This evidence supports PPE use by HCW, and especially N95 masks, to reduce the risk of a COVID-19 infection.

## 1 Introduction

For more than two years, the COVID-19 pandemic has challenged societies and healthcare systems worldwide. COVID-19 is transmitted by the SARS-CoV-2 virus (Severe Acute Respiratory Syndrome Coronavirus type 2), an 80- to 200-nm virus with a half-life of an estimated 1 h in air. Infected persons have the highest viral load in their salvia; however, viral RNA is also delectable in stool up to the 3rd and 4th week after the onset of symptoms [1]. Because the virus is mainly distributed by contact or droplet transmission [2], two main strategies used to combat the spread of the virus worldwide were social distancing and working from home-office, neither of which are feasible for healthcare workers (HCWs). In addition to these two social/physical distancing strategies, the World Health Organization (WHO) recommends maintaining thorough hand hygiene and using personal protective equipment (PPE) as parts of a comprehensive strategy to help HCWs by reducing viral transmission [3]. According to the WHO, proper PPE includes using gloves, gowns, goggles, face shield, respirators and masks [4] while providing healthcare [5]. Face masks, for example, allow the mechanical filtration of particles due to the weave of the fibres, whereby smaller particles are retained by the electrostatic properties of the fibres and diffusion characteristics [1]. However, the filtration capacity depends on the kind of face mask and on the fit on the face [6]. The use of hand hygiene, gloves and protective gowns should prevent viral spread by limiting the transmission of virus particles to and from objects or exposed skin [7]. The abilities of PPE users to put these on or take these off appropriately, as well as their waste management competencies, were also highlighted by the WHO as main infection prevention and control practice strategies [8].

This past year, several reviews have been published on the effectiveness of PPE in the clinical setting. Due to a lack of studies on COVID-19, these reviews included studies on different kind of respiratory infectious diseases like influenza [[9], [10], [11], [12], [13]]. However, these infectious diseases differ from COVID-19 with respect to the transmissibility of the virus, viral reproductive number and viral loads in the days of infection as well as in their signs and symptoms [14]. Therefore, only limited comparisons are possible.

Now, two years after the pandemic began, enough evidence should be available to assess the effectiveness of PPE, and specifically for the protection of HCWs from COVID-19. Although HCWs generally seem to accept PPE when caring for patients [15], possible side effects need to be considered when recommending their use. Regardless of the high acceptance rate, the healthcare workers need more information about how to properly handle PPE [16,17]. Furthermore, a qualitative study from the United Kingdom showed that the frequently changing and inconsistent information about the use of PPE contributes to incorrect handling and the infection of healthcare workers [18]. This clearly highlights the need for firm evidence on the topic of PPE use by HCWs.

Overall, these studies show that the use of PPE and hand hygiene are the main ways the spread of COVID-19 infections can be avoided among HCWs [3]. Moreover, several reviews have reported the effectiveness of PPE with regard to different kinds of respiratory infectious diseases. However, as these infectious diseases differ in various ways from COVID-19, only limited comparisons are possible. Therefore, this is the first study to specifically examine the spread of COVID-19 infections in connection with the currently recommended PPE.

Therefore, we assessed the effectiveness of PPE in terms of how well it protected HCWs from COVID-19 infections as well as the side effects experienced by HCWs who used PPE in clinical settings. In addition, we rated the certainty of these effects. The following research questions were addressed:

• How effective are different kinds of PPE in preventing COVID-19 infections in HCWs in the clinical setting?
• Is one kind of PPE more effective than another (e.g. different face masks)?
• Have any side effects of PPE been reported by the studies that examined their effectiveness?

## 2 Methods

We conducted a rapid review of the literature and meta-analyses by following a pre-defined protocol established by the Cochrane Rapid Reviews Methods Group [19]. This protocol was peer-reviewed and reflected upon by the authors of this paper. Scholars recommend conducting rapid reviews when evidence is needed quickly to address urgent and emergent healthcare topics [19], which is true for the current COVID-19 pandemic. Surgical face masks, cloth masks, N95 masks, gloves, gowns, goggles, face shields and air-purifying respirators were placed in the category PPE for the purposes of this review. We also included hand disinfection as one main intervention that potentially reduces the risk of a COVID-19 infection. To assess the evidence certainty, the GRADE method (Grading of Recommendations, Assessment, Development, and Evaluation method) was applied following the recommendations of the GRADE working group [20].

### 2.1 Search strategy

As recommended for rapid reviews [21], we conducted our review in two procedural stages. The first review process was conducted to identify the existing systematic reviews and to extract data from their included primary studies. The second review process was carried out to identify any further primary studies which had been missed or not included in the dataset.

To detect relevant systematic reviews, we searched the CDSR (Cochrane Database of Systematic Reviews), PubMed, CINAHL (Cumulative Index to Nursing and Allied Health Literature) and Epistemonikos. We searched for primary studies in CENTRAL (Cochrane Central Register of Controlled Trials), PubMed and CINAHL. No language restriction was set when carrying out these review processes. The studies had to have been published between January 1, 2020, i.e. at the onset of the COVID-19 pandemic, and June 24, 2021. The studies that met the following criteria were included in the final dataset:

- Study design: Published systematic reviews of intervention studies or observational studies and primary intervention or observational studies (cohort studies, case-control studies, case studies). Systematic reviews had to include a comprehensive review of the literature indexed in at least two databases and a quality appraisal of the included studies needed to have been performed and reported.
- Population: HCW who provide direct patient care.
- Intervention: Any type of PPE, including any kind of face masks, gloves, gowns, goggles, face shields and air-purifying respirators, special hand hygiene.
- Primary outcome: COVID-19 infection (clinical or laboratory-confirmed).
- Secondary outcome: Side effects, such as skin injuries, acceptance of PPE.
- Setting: Hospitals and long-term care institutions.

### 2.2 Exclusion criteria

We excluded systematic reviews of epidemiological or cross-sectional data, syntheses where no systematic approach was taken, reviews of guidelines or national recommendations, cross-sectional studies and qualitative studies. We also excluded studies that placed a focus on home care, outpatient care, primary care and HCWs who worked, e.g. as managers when the data were not separately displayed. In addition, we also excluded laboratory studies that did not involve humans.

A detailed description of the search strategy applied is provided in Supplementary Information A. Pairs of authors independently screened the titles, abstracts and full texts of the studies. Disagreement was resolved between the pairs of raters through discussion.

### 2.3 Data extraction and quality assessment

We extracted the following data from the reviews: author, publication year, review characteristics such as population, setting, intervention and outcomes as well as the search period and databases used. In addition, we extracted information on relevant, included primary studies. Regarding these primary studies, we extracted data on the study design, characteristics (e.g. population, intervention, setting, outcome) and results of interest (in the form of raw data, if possible).

The quality of the reviews was assessed by using a measurement tool to assess systematic reviews (AMSTAR II) [22], again independently by two reviewers. We decided to exclude the questions on the meta-analysis in AMSTART II due to the fact that we were interested in detecting primary relevant studies. We subsequently performed a new meta-analysis with studies identified as relevant.

The adapted Newcastle-Ottowa Scale (NOS) [23] was used to assess the risk of bias in the primary studies. We used the bias assessment from the included studies, if one was available. Otherwise, pairs of review authors independently assessed the risk of bias for each primary study. The NOS is a risk-of-bias assessment tool used for observational studies, including case-control and cohort studies, which is recommended by the Cochrane Collaboration [24]. The NOS assigns up to a maximum of nine points in three domains: 1) selection of study groups (four points); 2) comparability of groups (two points); and 3) ascertainment of exposure and outcomes (three points). Disagreement was resolved between the pairs of raters through discussion.

### 2.4 Data synthesis and certainty assessment

We referred to the Cochrane Handbook to guide the synthesis [24], grouping comparable interventions. If at least two comparable studies were available, results were pooled using the random-effects model. A random-effects assumption reflects the variation in the studies and the heterogeneity in the true effects that was estimated in each study [25]. Between-study heterogeneity was assessed using the I^2^ statistic (I^2^ > 60% and heterogeneity *P*-value < 0.05).

We calculated the pooled odds ratio (OR) with 95% confidence intervals (CI) using the Mantel-Haenszel method. Meta-analyses were performed with RevMan 5.4.1 [26].

The confidence in the evidence was determined for each PPE and the outcome of a COVID-19 infection using the GRADE method [27]. We used the GRADE considerations (risk of bias, inconsistency, indirectness, imprecision and other considerations) to assess the certainty of a body of evidence. This certainty can achieve between 1 and 4 levels, with level 1 being the lowest level, implying that the estimated effect has a very low certainty. A certainty level of four means that one can be highly confident that the true effect is similar to the estimated effect [28]. Observational studies are initially assigned the certainty level 2 (low certainty in the evidence), which can be either reduced due to the considerations mentioned but also increased, e.g. if a large effect is observed [28]. The certainty assessment was performed by the authors in consultation with one another, and decisions were transparently stated in the evidence profiles. GRADE profiles were created using the software GRADEpro GDT [20].

## 3 Results

**Fig. 1:**
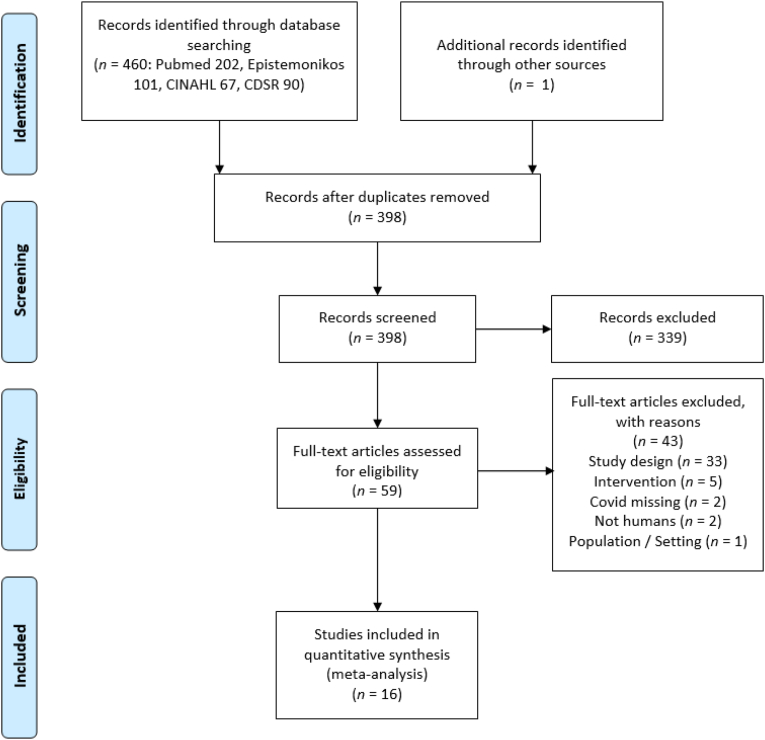
Flow chart of literature review of systematic reviews.

The systematic review resulted in the identification of 461 references for further screening in the first review process step. Of these references, we selected 59 for full-text screening. After screening these to determine if the eligibility criteria had been met, we excluded 43 articles. The remaining 16 systematic reviews included 11 different primary studies of interest. Fig. 1 displays the results of this literature review and the subsequent steps taken. Supplementary Information B displays a list of the excluded studies and the reason for their exclusion.

The second review process step, screening for further primary studies, yielded 208 hits of which six were identified as relevant. We screened the six full texts, but none of these met the inclusion criteria (Supplementary Information B).

### 3.1 Characteristics and quality assessment

Supplementary Information C and D display the characteristics and AMSTAR quality assessment of the included systematic reviews. Table 1 reports the characteristics of the included primary studies. Of the 11 included observational studies, three were prospective cohort studies [[29], [30], [31]], three were retrospective cohort studies [[32], [33], [34]], four were case control studies [[35], [36], [37], [38]], and one was a prospective case study [39]. The study participants in all HCWs working in a variety of occupations. One study also included non-HCWs [39]; however, the results were presented separately for HCWs and non-HCWs. Consequently, we were able to use these specific results. The study populations ranged from 37 [32] to 5442 participants [31], with the studies including a total of 9503 participants. The included studies were carried out to investigate different types of PPE (i.e. gloves, gowns, facemasks, hand hygiene, eye protection and thorough hand hygiene), different types of facemasks (i.e. surgical, N95 or not specified facemasks) and the presence of specific PPE regulations according to standard (e.g. WHO) versus no specific PPE regulations. None of the studies reported side effects associated with wearing PPE or the acceptance rates. The study quality ranged from three to seven out of nine stars possible (Table 1). Three of the included studies showed good overall quality [[35], [36], [37]]. We did not exclude studies based on the quality assessment.

**Table 1:** Characteristics of the included primary studies.

Authors (Year)	Included in following review	Study Design	Characteristics of the study				Quality of the study (NOS)		
			Population (n)	Setting	Intervention	Outcome measures	Selection	Comparability	Outcome
Barret et al. (2020)	Tian et al. (2020)	Prospective Cohort	Physicians, nurses, other HCW (*n* = 546)	University hospital	Gloves, Gown	Confirmed COVID-19 cases	✶✶	-	✶✶
Burke et al. (2020)	Chu et al. (2020)	Prospective case study	HCW (*n* = 163) and non-HCW (n = 175)^a^	Any health care setting (not specified)	Face masks, eye protection	Positive genetic markers of SARS-CoV-2 by RT-PCR	✶✶✶	-	✶
Chatterjee et al. (2020)	Tian et al. (2020)	Case control	Physicians, nurses, housekeeping staff, security guards, laboratory technician (*n* = 751)	Not specified	Gloves, Gown, face protection	Tested positive on qRT-PCR for SARS-CoV-2	✶✶✶	✶✶	✶✶
Chen et al. (2021)	Li et al. (2020)	Case control	Physicians, nurses, service assistants (*n* = 105)	Hospital	Masks (disposable non-surgical face mask, surgical mask or N95 mask)	Serological confirmed COVID-19 cases	✶✶✶	✶✶	✶✶
El-Boghdadly et al. (2020)	Ana et al. (2020)	Prospective cohort study	Anaesthesia, physician and other HCW involved in tracheal intubation (*n* = 1781)	Hospital (50% ICU)	PPE according to WHO minimum standard for aerosol-generating procedures	Incidence of COVID-19 infections or COVID-19 symptoms after tracheal intubation	✶✶		✶
Guo et al. (2020)	Li et al. (2020), Tian et al. (2020)	Case control	HCW not specified (*n* = 72)	Hospital (ICU, COVID-19 ward)	N95 respirator, hand hygiene	Confirmed SARS-CoV-2 transmission	✶✶✶✶	-	✶✶✶
Heinzerling et al. (2020)	Chu et al. (2020), Li et al. (2020), Tian et al. (2020)	Retrospective cohort	HCW not specified (*n* = 37)	Hospital	Gloves, Surgical mask	Laboratory confirmed COVID-19 infection	✶✶	-	✶✶
Ng et al. (2020)	Prashanth et al. (2020)	Case report	HCW not specified (*n* = 41)	Hospital	Surgical Mask, N95 mask	COVID-19 infections (symptoms, PCR)	✶	-	✶✶
Ran et al. (2020)	Tian et al. (2020)	Retrospective cohort	Physicians, nurses (*n* = 72)	Hospital	Hand hygiene, complete PPE (including masks, round caps, gloves, protective clothing, boot covers, goggles or face shields)	Diagnosed SARS-CoV-2 cases (RT-PCR)	✶✶	-	✶✶✶
Wang Q. et al. (2020a)	Chu et al. (2020)	Prospective Cohort	Surgeons, nurses (*n* = 5442)	Hospital	N95 mask	Confirmed COVID-19 infection (RT-PCR)	✶✶✶	-	✶✶
Wang X. et al. (2020b)	Chu et al. (2020), Li et al. (2020), Liang et al., 2020, Mingming et al. (2020)	Retrospective Cohort	Physicians, nurses (*n* = 493)	Hospital (neurosurgery)	N95 mask	Confirmed COVID-19 infections	✶✶	-	✶✶

HCW = health care worker, ICU = intensive care unit, PPE=Personal Protective Equipment, NOS= Newcastle Ottawa Scale.

a Only data on HCW was included.

### 3.2 Effect of PPE against COVID-19 infections

**Fig. 2:**
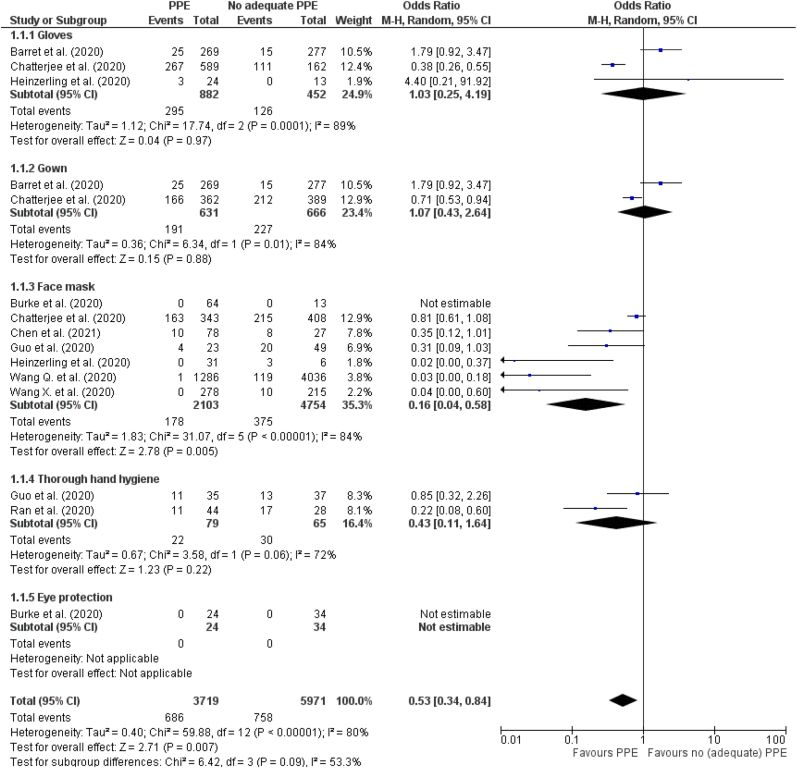
Meta-analysis comparing the protective effect of the use of PPE versus the non-adequate use of PPE against COVID-19 infection.

Nine studies compared the effectiveness of wearing PPE to not wearing adequate PPE by examining the COVID-19 infection rate in HCWs [29,[31], [32], [33], [34], [35], [36], [37],^39^]. Wearing PPE conferred significant protection against COVID-19 infection (OR = 0.53; 95% CI: 0.34–0.84; I^2^ = 80%), see Fig. 2. Table 2 shows the certainty assessment for each type of PPE.

**Table 2:** GRADE profile Protective Personal Equipment.

No. of studies	Study design	Certainty assessment					No. of participants		Effect		Certainty
		Risk of bias	Inconsistency	Indirectness	Imprecision	Others	PEE	No PPE	Relative [95% CI]	Absolute [95% CI]	
**Gloves**											
3	observational studies	not serious	serious^a^	not serious	serious^b^	none	295/882 (33.4%)	126/452 (27.9%)	OR 1.03 (0.25, 4.19)	**6 more per 1.000** (from 191 fewer to 339 more)	very low
**Gown**											
2	observational studies	not serious	serious^a^	not serious	serious^b^	none	192/631 (30.3%)	227/666 (34.1%)	OR 1.07 (0.43, 2.64)	**15 more per 1.000** (from 159 fewer to 236 more)	very low
**Face mask**											
7	observational studies	not serious	not serious^c^	not serious	not serious	strong association	178/2103 (8.5%)	375/4754 (7.9%)	OR 0.16 (0.04, 0.58)	**65 fewer per 1.000** (from 75 fewer to 32 fewer)	moderate
**Eye protection**											
1	observational study	serious^d^	not serious	not serious	very serious^e^	none	0/24 (0.0%)	0/34 (0.0%)	Not estimated		very low
**Thorough hand hygiene**											
2	observational studies	not serious	not serious^c^	not serious	serious^f^	none	22/79 (27.8%)	30/65 (46.2%)	OR 0.43 (0.11, 1.64)	**192 fewer per 1.000** (from 375 fewer to 123 more)	very low

Abbreviations: PPE = Protective Personal Equipment, CI = Confidence Interval.

a Heterogeneity >80%.

b Wide CI.

c High I^2^, but all studies favour intervention.

d Low quality according to Newcastle Ottawa Scale.

e Single study with small sample size.

f Wide CI, overlaps no-effect line.

Considering the results of the subgroup analysis, three studies [29,32,35] investigated the effectiveness of gloves and found no effect (OR = 1.09; 95% CI: 0.25–4.19). Due to the significant heterogeneity of the study results (I^2^ = 89%) and imprecise estimate in the effect, the certainty of this evidence was graded as very low.

Only two studies were carried out to investigate the effectiveness of wearing a gown on COVID-19 infections [29,35]. The results of the meta-analysis reveal no effect (OR = 1.07; 95% CI: 0.43–2.64) associated with the use of this type of PPE, with a very low certainty in the evidence.

Regarding the effectiveness of face masks, seven studies could be included. Results of the meta-analysis indicated that wearing face masks conferred significant protection against COVID-19 infection in the exposed HCWs (OR = 0.16; 95% CI: 0.04–0.58). Due to the large effect, the certainty of this evidence was graded as moderate (Table 2).

Two studies with low sample sizes (*n* = 144) [33,37] were conducted to assess the effectiveness of practicing thorough hand hygiene in protecting HCWs against a COVID-19 infection. Thorough hand hygiene showed a protective effect; however, the effect was not statistically significant (OR = 0.43; 95% CI: 0.11–1.64) and was graded down to a very low certainty due to the imprecise pooled result. One study assessing the effectiveness of using eye protection was included, but no COVID-19 infections occurred during the data collection [39].

### 3.3 Effects of using different kinds of face masks to protect against COVID-19 infection

Seven observational studies assessed the effectiveness of wearing face masks in protecting HCWs against COVID-19 infection [31,32,[34], [35], [36], [37],^39^].

**Fig. 3:**
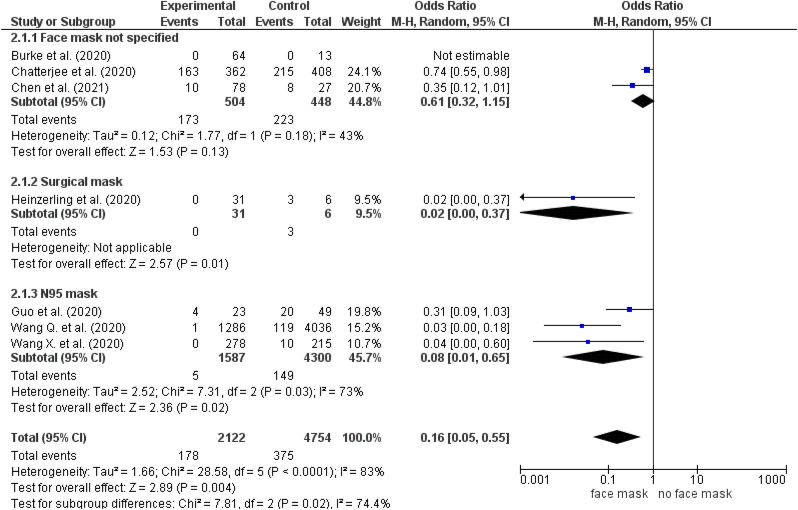
Meta-analysis comparing the protective effect of wearing face masks versus not wearing face masks against COVID-19 infection.

The overall effect showed that wearing face masks could significantly protect the HCWs from the infection (OR = 0.16; 95% CI: 0.05–0.55, I^2^ = 83%). Subgroup analyses were performed for unspecified face masks, surgical masks and N95 masks (Fig. 3).

The effect of wearing (unspecified) face masks on the outcome suggested that wearing a face mask may protect HCWs against the infection, but the overall effect was not statistically significant (OR = 0.61; 95% CI: 0.32–1.15). We rated the certainty of this effect as very low (Table 3) due to the imprecision with overlapping the no-effect line.

**Table 3:** GRADE profile face masks.

No. of studies	Study design	Certainty assessment					No. of participants		Effect		Certainty
		Risk of bias	Inconsistency	Indirectness	Imprecision	Others	Face mask	No mask	Relative (95% CI)	Absolute (95% CI)	
**Face mask not specified**											
3	observational studies	not serious	not serious	not serious	serious^a^	none	173/504 (34.3%)	223/448 (49.8%)	OR 0.61 (0.32–1.15)	**121 fewer per 1.000** (from 257 fewer to 35 more)	very low
**Surgical mask**											
1	observational studies	serious^b^	not serious	not serious	serious^c^	none	0/31 (0.0%)	3/6 (50%)	OR 0.02 (0.00–0.37)	**480 fewer per 1.000** (from 230 fewer to --)	very low
**N 95 mask**											
3	observational studies	not serious	not serious^d^	not serious	not serious	very strong association	5/1587 (0.3%)	149/4300 (3.5%)	OR 0.08 (0.01–0.65)	**32 fewer per 1.000** (from 34 fewer to 12 fewer)	high

Abbreviations: PPE = Protective Personal Equipment, CI = Confidence Interval.

a Wide CI, overlaps no-effect line.

b Low quality according to Newcastle Ottawa Scale.

c Single study with small sample size.

d High I^2^, but all studies favour intervention.

Only one study assessing the effectiveness of wearing surgical masks [32] was included in the subgroup analysis. This showed a statistically significant effect (OR = 0.02; 95% CI: 0.00–0.37) but very low confidence could be displayed in the evidence due to the small sample size.

Three studies were carried out to investigate the effects of wearing N95 masks on COVID-19 infection rates [31,34,37]. The estimate for the effect (OR = 0.08; 95% CI: 0.01–0.65) showed that wearing a N95 mask can protect HCWs from COVID-19 infection. Due to this large effect, this evidence was graded as high (Table 3). One study conducted an investigation of surgical masks as compared to N95 masks [38], but no cases of COVID-19 occurred in this study.

### 3.4 Effect of using proper PPE to protect against COVID-19 infection

**Fig. 4:**
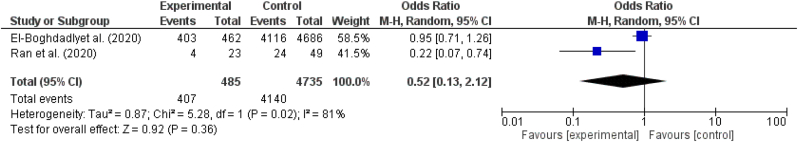
Meta-analysis comparing the protective effect of proper PPE use versus not using PPE properly against COVID-19 infection.

The WHO developed a guideline to prevent infection and control epidemic- and pandemic-prone acute respiratory infections in healthcare settings. With regard to COVID-19, they recommend the use of the following PPE: gloves, masks, goggles or face shields, and long-sleeved gowns with N95 respirators [4]. Two studies assessed the effectiveness of using proper PPE versus not using proper PPE on COVID-19 infection rates [30,33]. As compared to not using proper PPE, the use of proper PPE was seemed to protect HCWs against COVID-19 infection, but the effect was not statistically significant (OR = 0.52; 95% CI: 0.13–2.12) and a very low certainty could be displayed in this evidence (Fig. 4, Table 4).

**Table 4:** GRADE profile proper PPE.

No. of studies	Study design	Certainty assessment					No. of participants		Effect		Certainty
		Risk of bias	Inconsistency	Indirectness	Imprecision	Others	Proper PPE	No proper PPE	Relative (95% CI)	Absolute (95% CI)	
**Proper PPE**											
2	observational studies	serious^a^	not serious	not serious	serious^b^	none	407/485 (83.9%)	4140/4735 (87.4%)	OR 0.52 (0.13–2.12)	**91 fewer per 1.000** (from 399 fewer to 62 more)	very low

Abbreviations: PPE = Protective Personal Equipment, CI = Confidence Interval.

a Low quality of El-Boghdadlyet et al. according to Newcastle Ottawa Scale.

b Wide CI.

## 4 Discussion

In this rapid review and meta-analysis of data, we analysed how effectively PPE use in HCWs protected them against COVID-19 infection. Our results show that PPE and masks generally serves as significant protective factors against COVID-19 when worn properly. The subgroup analysis of the efficacy of using different types of masks indicated that wearing N95 masks significantly reduces the chance of being infected with SARS-CoV-2 with a high certainty. The data on surgical masks also indicated that they represented a significant protective factor. However, these data must be interpreted with caution, as they were extracted from a single study [32] and the certainty of the effect could only be rated as very low. Although the data indicate that the use of unspecified masks serves as a protective factor, the overall estimated effect was not significant, and the evidence was rated as having a very low certainty due to its imprecision.

We also explored the efficacy of applying further protective measures, such as practicing thorough hand hygiene and wearing a gown, but no significant effect was measured. This may be due to the small sample sizes in the studies on hand hygiene and the low number of included studies that analysed the use of gowns. Consequently, the certainty of the evidence was rated as very low.

No studies were identified that reported the side effects of wearing PPE. This may be due to the exclusion of cross-sectional studies and qualitative studies. We excluded cross-sectional studies, as they do not allow researchers to make causal inferences [40]. Moreover, the qualitative studies were also excluded, as we assumed that these could not measure our primary outcome “COVID-19 infections”. This may be why we did not find any side effect and acceptance results. Nevertheless, research on the side effects is available. Recently a systematic review of the impact of PPE on the physical health of HCWs was published by Ref. [41], who showed that 78% (42.8%–95.1%) of HCWs suffered from side effects of PPE use, such as dry skin, pressure injuries and headaches. In addition, they were able to verify a significant correlation between wearing PPE and skin reactions. The results of this systematic review, however, were all based on cross-sectional studies. This indicates that high quality study designs are needed when conducting research in the future on the side effects of wearing PPE. Similar results can be seen in a qualitative study of HCWs, in which the participants also complained of physical discomfort associated with PPE use, such as fatigue, skin reactions and headache [42].

None of the studies included in our review addressed the effects of correctly handling PPE. However, the WHO published guidelines which describe how to use different type of PPE correctly as well as the recommended wearing times [4]. In addition, temporary measures are described for use in a PPE shortage. Our review may not have enabled us to identify any results on PPE handling, because laboratory studies may be better suited for exploring this subject, and these were not included in this rapid review.

Due to a lack of evidence, our results do not allow us to comment on efficacy of different combinations of PPE. Nevertheless, the previously mentioned WHO guidelines recommend that HCWs wear different types of PPE, depending on their amount of exposure to SARS-CoV-2 and the procedures which are being carried out [43]. One included study examined the COVID-19 infection rates in HCWs after performing tracheal intubation on COVID-19-positive patients [30]. The authors compared the use of PPE according to the WHO minimum standard when performing aerosol-generating procedures and found that adhering to this standard offered no benefit as compared to wearing the PPE usually used to protect against COVID-19 infection. However, we note that the group which wore the usual protection was already wearing appropriate PPE and, therefore, no differences could be found between the groups.

### 4.1 Limitations

Our review was conducted according to a preliminary written protocol, which was peer-reviewed and reflected within the research group, but has not been published anywhere. However, since we conducted a rapid review, we decide not to publish the protocol. A further flaw of this study is the limitations of the study design. Excluding qualitative and cross-sectional studies kept us from finding results on PPE side effects and handling; for this reason, these should be included in the future when conducting research on this topic. We did not conduct a meta-regression to investigate the factors affecting heterogeneity, since only a small number of studies was available for each meta-analysis.

## 5 Conclusion

We found evidence that supports the use of PPE by HCW to reduce the risk of COVID-19 infection. Overall, our analytical results confirm the effectiveness of using personal protective equipment, and especially face masks, to protect against COVID-19 infection. With high certainty, our evidence indicates that using N95 masks significantly reduces the risk COVID-19 infection. Therefore, healthcare workers should strongly consider using these in the clinical setting. However, the certainty of evidence regarding different types of PPE and protective measures, such as thorough hand hygiene, remains low, indicating the need for further high-quality research studies.

## Author statements

The authors declare that there is no conflict of interest. As no personal, sensitive or confidential patient data were collected ethics approval was not required for this study. This research received no specific grant from any funding agency in the public, commercial, or not-for-profit sectors.

## Funding

This research did not receive any specific grant from funding agencies in the public, commercial, or not-for-profit sectors.

## Declaration of competing interest

The authors declare that they have no known competing financial interests or personal relationships that could have appeared to influence the work reported in this paper.
