# Supplementary material for: Rapid review and meta-analysis of the effectiveness of personal protective equipment for healthcare workers during the COVID-19 pandemic
Source: Public Health Pract (Oxf). 2022 Jun 13;4:100280. doi: 10.1016/j.puhip.2022.100280 (PMC9190185; doi:10.1016/j.puhip.2022.100280)
Supplement: Multimedia component 1 [file mmc1.docx]

**Supplementary Appendices**

## Supplementary data A: review strategy

## Supplementary data B: excluded studies

## Supplementary data C: characteristics of the included systematic reviews

## Supplementary data D: AMSTAR quality assessment of the included systematic reviews

Supplementary Information A: review strategy

Table 1. Search terms used for literature review

| **Intervention** | **Outcome** | **Population** |
| --- | --- | --- |
| **Personal protective equipment:** Protective clothing, gown, coverall, protective layer*, surgical toga, apron or smock, hazmat suit, PPE, personal protective equipment or safety equipment or safety gear  **Masks:** Masks, respiratory protective devices, respiratory protective devices, mask*, face mask*, facemask*, respiratory protection, respirator, FFP2, FFP3, FFP, N95, N 95, PAPR, air purifying respirator, filtering face piece, facial protection equipment, surgical hood, hood, medical mask, cloth mask*, conventional mask, home-made mask, home-made face mask  **Eye-/facial protection:** eye protective device*, transparent panel, visor, goggle*, facial protection equipment, safety glass*, safety spectacles, face shield, face proactive device*, eye shield*, aerosol face protection | **Infection control:** Equipment Contamination, infection control*, protective measure*, safety measures, safety precaution*, airborne precaution*, decontaminat*, resanitiz*, resanitis*, desanitisat*, contaminat*, antisept, biocid*, steriliz*, sanitiz*, bleach*  **Covid-19:** Coronavirus, SARS CoV-2, Corona, Covid-19, 2019-novel-corona*, 2019-new-corona, novel-corona, new-corona, 2019-CoV, 2019-nCoV, nCoV, coronavirus disease-2019, SARS2, SARS-2, 2019ncov, coronavirus-2 | **Healthcare workers:** nursing, nursing personnel, nursing staff, healthcare worker, health personnel, personnel, Hospital, health provider*, medical worker*, medical personnel, medical professiona*l, nurses, nurses aides, HCW* |

Table 2. Search strings used to search the databases (exemplary for systematic reviews)

| **Database** | **Search string** |
| --- | --- |
| Cochrane Database of Systematic Reviews, Epistemonikos | #1 MeSH descriptor: [Protective Clothing] explode all trees  #2 gown OR coverall OR protective layer* OR surgical toga OR apron OR smock* OR hazmat suit OR PPE OR Personal protective equipment OR safety equipment OR safety gear  #3 MeSH descriptor: [Respiratory Protective Devices] explode all trees  #4 MeSH descriptor: [Personal Protective Equipment] explode all trees  #5 MeSH descriptor: [N95 Respirators] explode all trees  #6 MeSH descriptor: [Masks] explode all trees  #7 respiratory protective devices OR mask* OR face mask* OR facemask* OR respiratory protection OR respirator* OR FFP2 OR FFP3 OR FFP OR N95 OR N 95 OR PAPR OR air purifying respirator OR filtering face piece OR facial protection equipment OR surgical hood OR hood OR medical mask OR cloth mask* OR conventional mask OR home-made mask OR home-made face mask  #8 MeSH descriptor: [Eye Protective Devices] explode all trees  #9 Transparent panel OR visor OR goggle* OR facial protection equipment OR safety glass* OR safety spectacles OR face shield* OR face proactive device* OR eye shield* OR aerosol face protection  #10 #1 OR #2 OR #3 OR #4 OR #5 OR #6 OR #7 OR #8 OR #9  #11 MeSH descriptor: [COVID-19] explode all trees  #12 MeSH descriptor: [SARS-CoV-2] explode all trees  #13 Coronavirus OR SARS-CoV-2 OR Corona OR Covid-19 OR novel-corona OR new-corona OR nCoV OR SARS2 OR SARS-2 OR coronavirus-2  #14 #11 OR #12 OR #13  #15 MeSH descriptor: [Nursing] explode all trees  #16 MeSH descriptor: [Health Personnel] explode all trees  #17 MeSH descriptor: [Medical Staff] explode all trees  #18 #15 OR #16 OR #17  #19 Nurs* OR nursing personnel OR nursing staff OR healthcare worker OR health personnel OR health provider OR medical worker OR medical personnel OR medical professional OR health care professional OR nurses OR nurses aides OR HCW*  #20 #18 OR #19  #21 MeSH descriptor: [Equipment Contamination] explode all trees  #22 MeSH descriptor: [Infection Control] explode all trees  #23 MeSH descriptor: [Infections] explode all trees  #24 protective measur OR safety measure OR safety precaution OR airborne precaution* OR infection control* OR decontaminat* OR resanitiz* OR desaniti* OR contaminat* OR antisept* OR biocid* OR steriliz* OR sanitize* OR bleach*  #25 #21 OR #22 OR #23 OR #24  #26 #10 AND #14 AND #20 AND #25 |
| PubMed | #19 Search: #8 AND #11 AND #14 AND #17 Filters: Systematic Review  #18 Search: #8 AND #11 AND #14 AND #17  #17 Search: #15 OR #16  #16  Search: protective measur OR safety measure OR safety precaution OR airborne precaution* OR infection control* OR decontaminat* OR resanitiz* OR desaniti* OR contaminat* OR antisept* OR biocid* OR steriliz* OR sanitize* OR bleach*  #15 Search: (("Equipment Contamination"[Mesh]) OR "Infection Control"[Mesh]) OR "Infections"[Mesh] Sort by: Most Recent  #14 Search: #12 OR #13  #13  Search: Nurs* OR nursing personnel OR nursing staff OR healthcare worker OR health personnel OR health provider OR medical worker OR medical personnel OR medical professional OR health care professional OR nurses OR nurses aides OR HCW*  #12  Search: (("Nurses"[Mesh] OR "Nurse Practitioners"[Mesh] OR "Nurse Clinicians"[Mesh] OR "Nurse Specialists"[Mesh]) OR ( "Health Personnel"[Mesh] OR "Allied Health Personnel"[Mesh] )) OR ( "Medical Staff"[Mesh] OR "Medical Staff, Hospital"[Mesh] ) Sort by: Most Recent  #11 Search: #9 OR #10  #10  Search: Coronavirus OR SARS CoV-2 OR Corona OR Covid-19 OR 2019-novel-corona* OR novel-corona OR new-corona OR 2019-CoV OR 2019-nCoV OR nCoV OR coronavirus disease OR SARS2 OR SARS-2 OR 2019ncov OR coronavirus-2  #9 Search: "COVID-19"[Mesh] OR "SARS-CoV-2"[Mesh] Sort by: Most Recent  #8 Search: #1 OR #2 OR #4 OR #5 OR #6 OR #7  #7  Search: Transparent panel OR visor OR goggle* OR facial protection equipment OR safety glass* OR safety spectacles OR face shield* OR face proactive device* OR eye shield* OR aerosol face protection  #6 Search: "Eye Protective Devices"[Mesh] Sort by: Most Recent  #5  Search: respiratory protective devices OR mask* OR face mask* OR facemask* OR respiratory protection OR respirator* OR FFP2 OR FFP3 OR FFP OR N95 OR N 95 OR PAPR OR air purifying respirator OR filtering face piece OR facial protection equipment OR surgical hood OR hood OR medical mask OR cloth mask* OR conventional mask OR home-made mask OR home-made face mask  #4  Search: ("Respiratory Protective Devices"[Mesh] OR "Personal Protective Equipment"[Mesh] OR "N95 Respirators"[Mesh]) OR "Masks"[Mesh] Sort by: Most Recent  #2  Search: gown OR coverall OR protective layer* OR surgical toga OR apron OR smock* OR hazmat suit OR PPE OR Personal protective equipment OR safety equipment OR safety gear  #1  Search: protective clothing[MeSH Major Topic] |
| CINAHL | S6 S1 AND S2 AND S3 AND S4 AND S5  S5 ( (MH "COVID-19") OR (MH "SARS Virus") OR (MH "Severe Acute Respiratory Syndrome") ) OR ( ( (MH "COVID-19") OR (MH "SARS Virus") OR (MH "Severe Acute Respiratory Syndrome") ) OR ( Coronavirus OR SARS CoV-2 OR Corona OR Covid-19 OR 2019-novel-corona* OR novel-corona OR new-corona OR 2019-CoV OR 2019-nCoV OR nCoV OR coronavirus disease OR SARS2 OR SARS-2 OR 2019ncov OR coronavirus-2 ) )  S4 (TI (systematic* n3 review*)) or (AB (systematic* n3 review*)) or (TI (systematic* n3 bibliographic*)) or (AB (systematic* n3 bibliographic*)) or (TI (systematic* n3 literature)) or (AB (systematic* n3 literature)) or (TI (comprehensive* n3 literature)) or (AB (comprehensive* n3 literature)) or (TI (comprehensive* n3 bibliographic*)) or (AB (comprehensive* n3 bibliographic*)) or (TI (integrative n3 review)) or (AB (integrative n3 review)) or (JN “Cochrane Database of Systematic Reviews”) or (TI (information n2 synthesis)) or (TI (data n2 synthesis)) or (AB (information n2 synthesis)) or (AB (data n2 synthesis)) or (TI (data n2 extract*)) or (AB (data n2 extract*)) or (TI (medline or pubmed or psyclit or cinahl or (psycinfo not “psycinfo database”) or “web of science” or scopus or embase)) or (AB (medline or pubmed or psyclit or cinahl or (psycinfo not “psycinfo database”) or “web of science” or scopus or embase)) or (MH “Systematic Review”) or (MH “Meta Analysis”) or (TI (meta-analy* or metaanaly*)) or (AB (meta-analy* or metaanaly*))  S3 ( (MH "Equipment Contamination") OR (MH "Infection Control") OR (MH "Infection Control (Iowa NIC)") OR (MH "Infection") ) OR ( protective measur OR safety measure OR safety precaution OR airborne precaution* OR infection control* OR decontaminat* OR resanitiz* OR desaniti* OR contaminat* OR antisept* OR biocid* OR steriliz* OR sanitize* OR bleach* )  S2 ( (MH "Nurses") OR (MH "Health Personnel") OR (MH "Medical Staff") OR (MH "Medical Staff, Hospital") OR (MH "Nursing Staff, Hospital") ) OR ( Nurs* OR nursing personnel OR nursing staff OR healthcare worker OR health personnel OR health provider OR medical worker OR medical personnel OR medical professional OR health care professional OR nurses OR nurses aides OR HCW* )  S1 ( (MH "Protective Clothing") OR (MH "Respiratory Protective Devices") OR (MH "Personal Protective Equipment") OR (MH "Infection Protection (Iowa NIC)") OR (MH "Gloves") OR (MH "Masks") ) OR (MH "Eye Protective Devices") OR ( ( gown OR coverall OR protective layer* OR surgical toga OR apron OR smock* OR hazmat suit OR PPE OR Personal protective equipment OR safety equipment OR safety gear ) OR ( respiratory protective devices OR mask* OR face mask* OR facemask* OR respiratory protection OR respirator* OR FFP2 OR FFP3 OR FFP OR N95 OR N 95 OR PAPR OR air purifying respirator OR filtering face piece OR facial protection equipment OR surgical hood OR hood OR medical mask OR cloth mask* OR conventional mask OR home-made mask OR home-made face mask ) OR ( Transparent panel OR visor OR goggle* OR facial protection equipment OR safety glass* OR safety spectacles OR face shield* OR face proactive device* OR eye shield* OR aerosol face protection ) ) |

Supplementary Information B: excluded studies

Excluded systematic reviews

| **Reasons for exclusion: systematic reviews** | | | | | | | |
| --- | --- | --- | --- | --- | --- | --- | --- |
| **Nr.** | **Study** | **Design not fitting** | **Intervention not fitting** | **COVID missing** | **Population/Setting not fitting** | **No humans included** | **Reason for exclusion** |
| 1 | Abbas, 2020 | **1** | 0 | 0 | 0 | 0 | Design not fitting |
| 2 | Agarwal, 2021 | 0 | **1** | 0 | 0 | 0 | Intervention not fitting |
| 3 | Allessandra Rocha, 2020 | **1** | 0 | 0 | 0 | 0 | Design not fitting |
| 4 | Arias-López, 2020 | **1** | 0 | 0 | 0 | 0 | Design not fitting |
| 5 | Baldock, 2020 | **1** | 0 | 0 | 0 | 0 | Design not fitting |
| 6 | Boškoski, 2020 | **1** | 0 | 0 | 0 | 0 | Design not fitting |
| 7 | Boyce, 2020 | **1** | 0 | 0 | 0 | 0 | Design not fitting |
| 8 | Christopher, 2020 | **1** | 0 | 0 | 0 | 0 | Design not fitting |
| 9 | Elizarrarás-Rivas, 2020 | **1** | 0 | 0 | 0 | 0 | Design not fitting |
| 10 | Felipe G., 2020 | **1** | 0 | 0 | 0 | 0 | Design not fitting |
| 11 | Gnatta, 2020 | **1** | 0 | 0 | 0 | 0 | Design not fitting |
| 12 | GÖG, 2020 | **1** | 0 | 0 | 0 | 0 | Design not fitting |
| 13 | Gomes, 2020 | **1** | 0 | 0 | 0 | 0 | Design not fitting |
| 14 | Gross, 2020 | 0 | **1** | 0 | 0 | 0 | Intervention not fitting |
| 15 | Ha, 2020 | **1** | 0 | 0 | 0 | 0 | Design not fitting |
| 16 | Hirschmann, 2020 | **1** | 0 | 0 | 0 | 0 | Design not fitting |
| 17 | Isaacs, 2020 | **1** | 0 | 0 | 0 | 0 | Design not fitting |
| 18 | Jefferson, 2020 | 0 | 0 | **1** | 0 | 0 | COVID not included in search strategy |
| 19 | Jessop, 2020 | **1** | 0 | 0 | 0 | 0 | Design not fitting |
| 20 | Kaur, 2020 | **1** | 0 | 0 | 0 | 0 | Design not fitting |
| 21 | Kumbargere Nagraj, 2020 | 0 | **1** | 0 | 0 | 0 | Intervention not fitting no PPE |
| 22 | Luqman-Arafath, 2020 | 0 | **1** | 0 | 0 | 0 | Intervention not fitting |
| 23 | MacIntyre, 2020 | **1** | 0 | 0 | 0 | 0 | Design not fitting |
| 24 | Medina Garzón, 2020 | **1** | 0 | 0 | 0 | 0 | Design not fitting |
| 25 | Rosario Martinho, 2020 | **1** | 0 | 0 | 0 | 0 | Design not fitting |
| 26 | Marasinghe, 2020 | 0 | 0 | 0 | **1** | 0 | Setting not fitting |
| 27 | Misbah, 2020 | **1** | 0 | 0 | 0 | 0 | Design not fitting |
| 28 | Moslehi, 2020 | **1** | 0 | 0 | 0 | 0 | Design not fitting |
| 29 | Neto, 2020 | **1** | 0 | 0 | 0 | 0 | Design not fitting |
| 30 | Noorimotlagh, 2020 | **1** | 0 | 0 | 0 | 0 | Design not fitting |
| 31 | O'Hearn, 2020 | 0 | 0 | 0 | 0 | **1** | Only laboratory studies |
| 32 | O'Hearn, 2020 | 0 | 0 | 0 | 0 | **1** | Only laboratory studies |
| 33 | Offeddu, 2017 | 0 | **1** | 0 | 0 | 0 | Intervention not fitting |
| 34 | Perrone, 2021 | **1** | 0 | 0 | 0 | 0 | Design not fitting |
| 35 | Rahmidha, 2020 | **1** | 0 | 0 | 0 | 0 | Design not fitting |
| 36 | Samaranayake, 2020 | 0 | 0 | **1** | 0 | 0 | No focus on COVID |
| 37 | San Martín-Rodríguez, 2021 | **1** | 0 | 0 | 0 | 0 | Design not fitting |
| 38 | Santy Irene, 2020 | **1** | 0 | 0 | 0 | 0 | Design not fitting |
| 39 | Schnitzbauer, 2020 | **1** | 0 | 0 | 0 | 0 | Design not fitting |
| 40 | Seresirikachorn, 2021 | **1** | 0 | 0 | 0 | 0 | Design not fitting |
| 41 | Steinberg, 2020 | **1** | 0 | 0 | 0 | 0 | Design not fitting |
| 42 | Thapa, 2021 | **1** | 0 | 0 | 0 | 0 | Design not fitting |
| 43 | Wang, 2020 | **1** | 0 | 0 | 0 | 0 | Design not fitting |
|  | **Total:** | **33** | **5** | **2** | **1** | **2** |  |

Excluded primary studies

|  | | | | | |
| --- | --- | --- | --- | --- | --- |
| **Reasons for exclusion: primary studies** | | | | | |
| **Nr.** | **Study** | **Design not fitting** | **Intervention not fitting** | **Outcome not fitting** | **Reason for exclusion** |
| 1 | Gasparino et al. (2021) | 0 | **1** | 0 | Intervention not fitting |
| 2 | Li et al. (2020) | **1** | 0 | 0 | Design not fitting |
| 3 | MacIntyre et al. (2020) | 0 | 0 | **1** | Outcome not fitting |
| 4 | Rashed et al. (2020) | 0 | 0 | **1** | Outcome not fitting |
| 5 | Williams et al. (2021) | 0 | 0 | **1** | Outcome not fitting |
| 6 | Zhang et al. (2021) | 0 | 0 | **1** | Outcome not fitting |
|  | **Total:** | **1** | **1** | **4** |  |

**Supplementary Information C: characteristics of the included systematic reviews**

| **Authors (Year)** | **Review characteristics (according to review inclusion criteria)** | | | | | |
| --- | --- | --- | --- | --- | --- | --- |
|  | **Interventions of interest** | **Endpoints of interest** | **Population of interest** | **Setting of interest** | **Amstar Overall Rating** | **Included studies of interest* (References)** |
| **Ana et al. (2020)** | Powered Air-Purifying  Respirator (PAPR) studied separately or within a Personal Protective Equipment (PPE) | - HCW infection rates - compliance with guidance on use of PAPR - level of wearer comfort whilst   using the PAPR;   - objective and/or subjective measures of work of breathing during the use of PAPR; - impact of structured training programs on PAPR use; | Healthcare workers | Inpatient care/Critical  Care/Intensive Care; | 10 | - El-Boghdadly et al. (2020) (prospective cohort study) |
| **Bakhit et al. (2021)** | Face masks | Compliance, discomforts, harms, adverse events | People of any age and gender including HCW | Any setting (including healthcare setting) | 11 | No studies on Covid-19 |
| **Bartoszko et al. (2020)** | Medical masks vs. N95 respirators | Preventing laboratory  confirmed  viral infection and respiratory illness including coronavirus | Healthcare workers | All health care settings | 8 | No Covid-19 studies |
| **Chu et al. (2020)** | Physical distancing, face masks, and eye protection | Person-to-person transmission of SARS-CoV-2 and  COVID-19  comparing distances between people and  COVID-19 infected patients | Health-care and non-health-care (eg. community) | Healthcare and non-healthcare setting | 9 | - Burke et al. (2020) (prospective case study) - Heinzerling et al. (2020) (case-control/retrospective cohort-study) - Wang X. et al. (2020) (prospective cohort study) - Wang Q. et al. (2020) (prospective cohort study) |
| **Iannone et al. (2020)** | N95 respirators vs. surgical masks | - SARS-CoV-2 infection - Clinical respiratory illness - Influenza like illness - Laboratory-confirmed respiratory viral infection - Laboratory-confirmed bacterial colonization - Laboratory-confirmed respiratory infection - Laboratory-confirmed influenza - Discomfort of wearing respiratory protections | HWCs exposed to SARS-CoV-2 or any other respiratory infection | In-patient and out-patient hospital setting | 7 | No Covid-19 studies |
| **Li et al. (2020)** | Face masks | Laboratory confirmed SARS-CoV-2 infection | Healthcare workers and non-healthcare workers | Any setting | 9 | - Chen et al. (2021) (case- control study) - Guo et al. (2020) (case-control study) - Heinzerling et al. (2020) (case-control/retrospective cohort-study) - Wang X. et al. (2020) (case-control/retrospective cohort-study) |
| **Liang et al. (2020)** | Face masks | Laboratory confirmed  respiratory virus transmission | HCW and non-HCW | Any setting | 8 | - Wang X. et al. (2020) (case-control/retrospective cohort-study) |
| **Mahdi et al. (2020)** | Additional measures for the management of potentially infectious persons visiting a dental practice: Patient Triage Prior to Patient Arrival; Patient Evaluation and Screening Upon Arrival; Infection Control during Dental Treatment (Hand hygiene, mouth rinse, masks – FFP1/surgical masks); disinfection after treatment | Management of infection control procedures through additional measures during the pandemic | Dental professional HCWs | Dental health care settings | 6 | No studies on Covid-19 |
| **Mingming et al. (2020)** | Facemasks (surgical masks, N95) | Virus transmission, preventing respiratory viral infection | HCW, Non-HCW | any setting including HCS | 6 | - Wang X. et al. (2020) (case-control/retrospective cohort-study) |
| **Prashanth et al. (2020)** | Surgical masks versus respirators | SARS-CoV-2 protection | HCW delivering secondary care, | In -Patient | 8 | - Ng et al. (2020) (case report) |
| **Santos et al. (2020)** | Homemade and/or commercial cloth mask, surgical mask and/or N95 respirator | Reducing contamination and transmission | HCW | Not stated | 8 | No studies on Covid-19 |
| **Sharma et al. (2020)** | Cloth face mask | Reducing the risk of contracting viral infections | HCW | Clinical and community setting | 6 | No studies on Covid-19 |
| **Tian et al. (2020)** | Personal protective equipment (PPE) use (e.g. surgical mask, N95 respirator or equivalent, gowns, full-body protection, eye and face protection, gloves, proper donning and doffing techniques), hand hygiene | Protective effects of infection prevention and control | Infected and non-infected HCW | Healthcare setting | 8 | - Wang X. (2020) (Retrospective Cohort) - Barret et al. (2020) (prospective cohort study) - Chatterjee et al. (2020) (case control) - Wang Q et al. (2020) (prospective cohort study) - Ran et al. (2020) (Retrospective Cohort) - Heinzerling et al. (2020) (Retrospective Cohort) - Guo et al. (2020) (Case control) |
| **Verbeek et al. (2020)** | Full-body protection (PPE)  such as gowns, coveralls, or hazardous materials (hazmat) suits;  eye and face protection such as glasses, goggles, face shields or  visors, or masks or hoods that cover the entire head; hand protection: gloves; and foot protection: overshoes or boots. | - Contamination - Infection - Compliance with guidance   2. Outcome  User-reported assessment of comfort and convenience  Costs or resource use   - Time to don and do. the PPE | Simulation studies: volunteers or HCW; field studies HCW or ancilliary staff | Exposed to EVD, SARS or COVID-19 | 10 | No studies on Covid-19 |
| **Yaacoub et al. (2020)** | PPE | Risk of COVID-19 transmission | HCW | Any setting | 10 | No studies on Covid-19 |
| **Yin et al. (2020)** | Respiratory protective equipment (surgical mask, cloth mask, N95 mask) | Incidence of  laboratory-confirmed viral respiratory infection | HCW | Healthcare settings worldwide | 6 | No studies on Covid-19 |

## Supplementary Information D: AMSTAR quality assessment of the included systematic reviews

| **Author** | **Question and inclusion** | **Protocol** | **Study design** | **Comprehensive Search** | **Study selection** | **Data Extraction** | **Excluded studies: justification** | **Included Studies Details** | **Risk of Bias** | **Funding Sources** | **Conflict of Interest** | **Total** |
| --- | --- | --- | --- | --- | --- | --- | --- | --- | --- | --- | --- | --- |
| **Ana, 2020** | Yes | Partial Yes | Yes | Yes | Yes | Yes | Partial Yes | Partial Yes | Yes | No | Yes | **10** |
| **Bakhit, 2021** | Yes | Yes | Yes | Yes | Yes | Yes | Yes | Partial Yes | Yes | Yes | Yes | **11** |
| **Bartoszko, 2020** | Yes | No | No | No | Yes | Yes | Partial Yes | Partial Yes | Yes | Yes | Yes | **8** |
| **Chu, 2020** | No | Yes | No | Yes | Yes | Yes | Partial Yes | Partial Yes | Yes | Yes | Yes | **9** |
| **Iannone, 2020** | Yes | No | No | No | Yes | Yes | Yes | Yes | Yes | No | Yes | **7** |
| **Li, 2020** | Yes | Yes | No | Partial Yes | Yes | Yes | Partial Yes | Partial Yes | Yes | No | Yes | **9** |
| **Liang, 2020** | Yes | No | No | Partial Yes | Yes | Yes | Partial Yes | Partial Yes | Yes | No | Yes | **8** |
| **Mahdi, 2020** | No | No | No | Partial Yes | Yes | No | Partial Yes | Partial Yes | Yes | No | Yes | **6** |
| **Mingming, 2020** | Yes | No | No | Partial Yes | Yes | No | No | Yes | Yes | No | Yes | **6** |
| **Prashanth, 2020** | Yes | No | Yes | Yes | Yes | Yes | No | Yes | Yes | No | Yes | **8** |
| **Santos, 2020** | Yes | Partial Yes | No | No | Yes | Yes | Yes | Partial Yes | Yes | No | Yes | **8** |
| **Sharma, 2020** | Yes | No | No | Partial Yes | Yes | No | No | Partial Yes | Yes | No | Yes | **6** |
| **Tian, 2020** | Yes | Partial Yes | No | No | Yes | Yes | Partial Yes | Partial Yes | Yes | No | Yes | **8** |
| **Verbeek, 2020** | Yes | Yes | Yes | Partial Yes | Yes | Yes | Partial Yes | Yes | Yes | Yes | No | **10** |
| **Yaacoub, 2020** | Yes | Yes | No | Yes | Yes | Yes | Partial Yes | Partial Yes | Yes | Yes | Yes | **10** |
| **Yin, 2020** | Yes | Partial Yes | No | Partial Yes | No | Yes | No | No | Yes | No | Yes | **6** |
